# Surgical Surprise: Cutaneous Metastasis Presenting for Mohs Micrographic Surgery Without a Prior Diagnostic Biopsy

**DOI:** 10.2196/33241

**Published:** 2021-11-01

**Authors:** Spencer Dunaway, Pushkar Aggarwal, Cristin Shaughnessy, Scott Neltner

**Affiliations:** 1 Department of Dermatology University of Cincinnati College of Medicine Cincinnati, OH United States; 2 University of Cincinnati College of Medicine Cincinnati, OH United States

**Keywords:** cutaneous metastasis, Mohs surgery, biopsy, micrographic surgery, dermatology, dermatologist, skin cancer, melanoma

## Case Report

A 60-year-old Caucasian woman presented for Mohs micrographic surgery (MMS) after being referred for a clinically presumed basal cell carcinoma of the scalp. Three months prior, while living abroad, the patient developed a nodule on the vertex of her scalp. The patient’s primary care physician initially treated the lesion as a cyst with antibiotics. However, after no improvement, she received a second opinion and was told it was a basal cell carcinoma. The patient returned to the United States for treatment and was seen by a plastic surgeon, who subsequently referred her to a Mohs micrographic surgeon.

**Figure 1:**
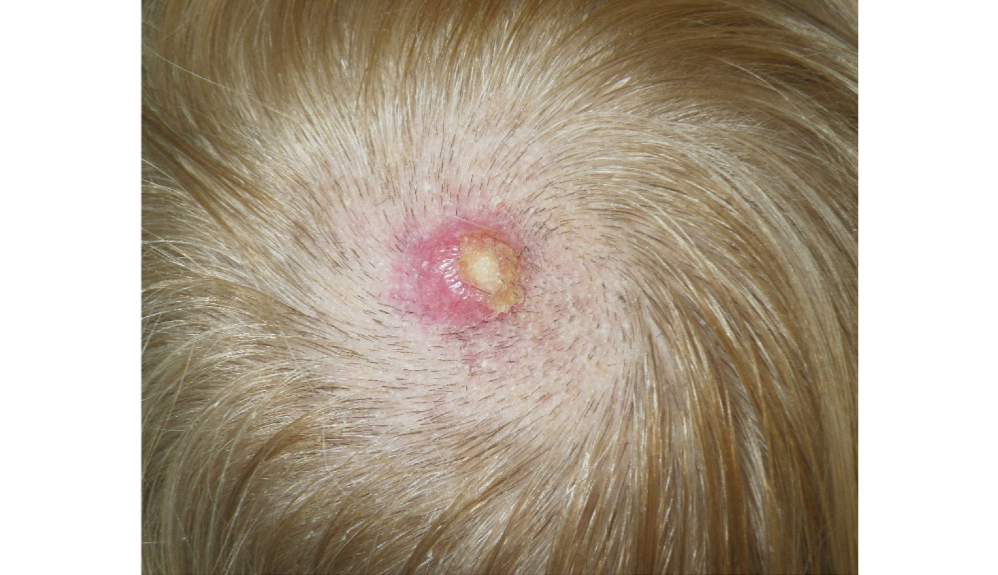
A 1.7-cm violaceous, slightly scaly nodule at the vertex of the patient’s scalp, revealed through preoperative evaluation.

Upon presentation, the patient had a 1.7-cm violaceous, slightly scaly nodule on the vertex of the scalp (Figure 1).

The patient also complained of a tender subcutaneous nodule on her right flank that was noted after a bicycle accident, also approximately 3 months prior. Pertinent history included 10 pack-years of smoking in her 20s. Prior to initiating Mohs surgery, a shave biopsy was performed, revealing a basaloid proliferation with adenomatous differentiation diffusely involving the dermis and subcutis. Given the diagnostic ambiguity regarding this lesion, it was sent for permanent section processing.

**Figure 2:**
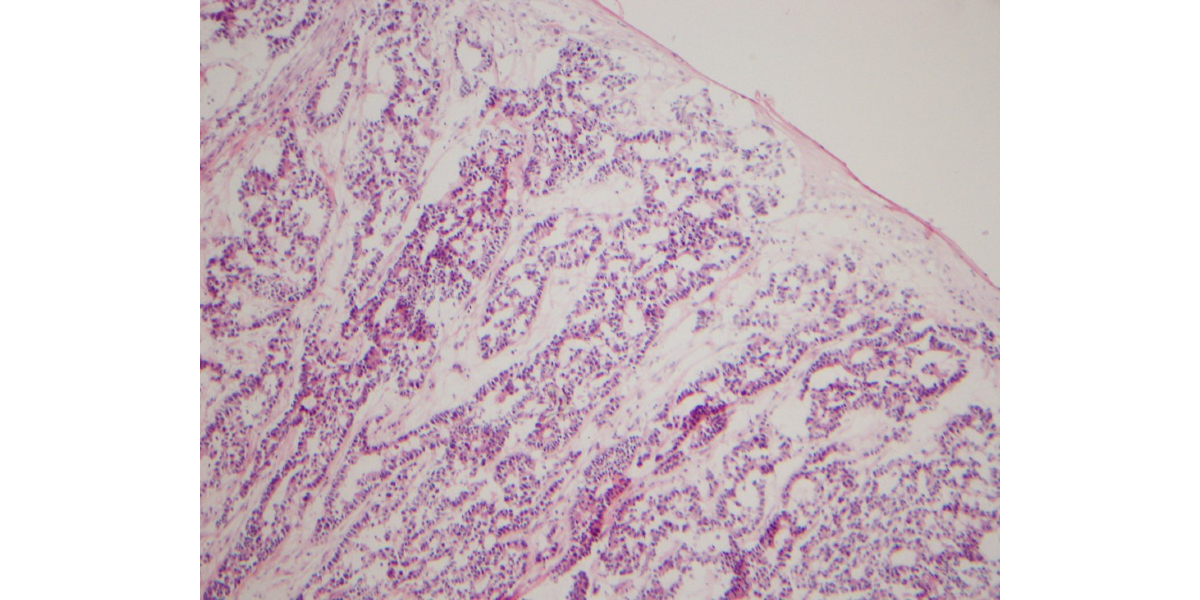
Frozen section with basaloid proliferation with adenomatous differentiation.

**Figure 3:**
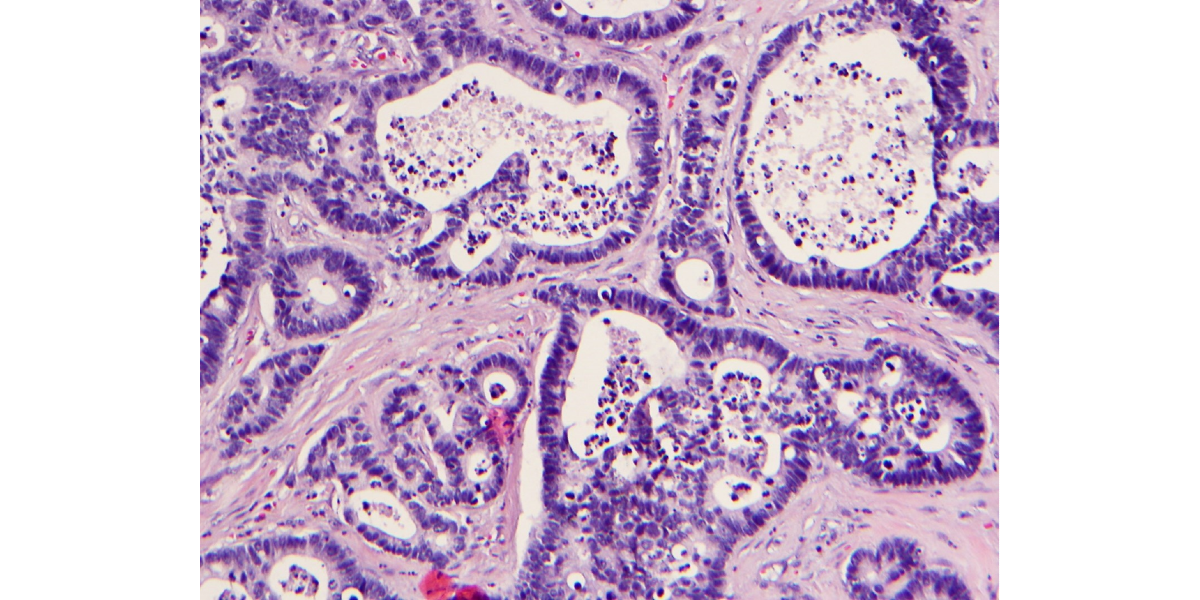
Permanent section with proliferation of variably shaped and sized islands with a central lumen lined by crowded, hyperchromatic, and large columnar cells with many atypical mitotic figures.

**Figure 4:**
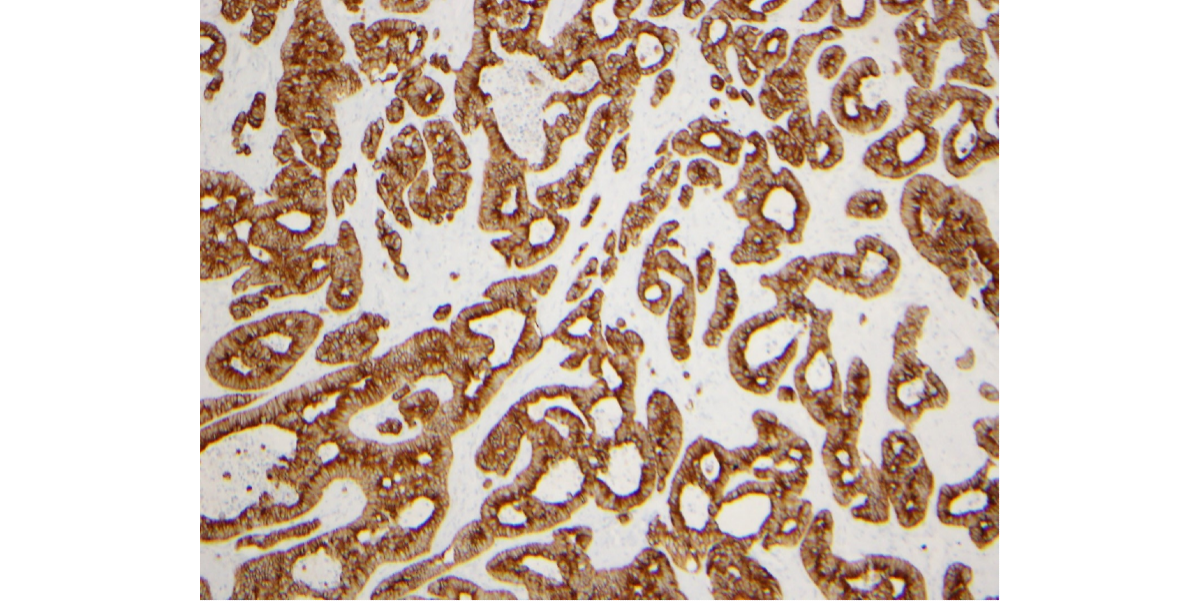
CK7 (cytokeratin 7) positive.

**Figure 5:**
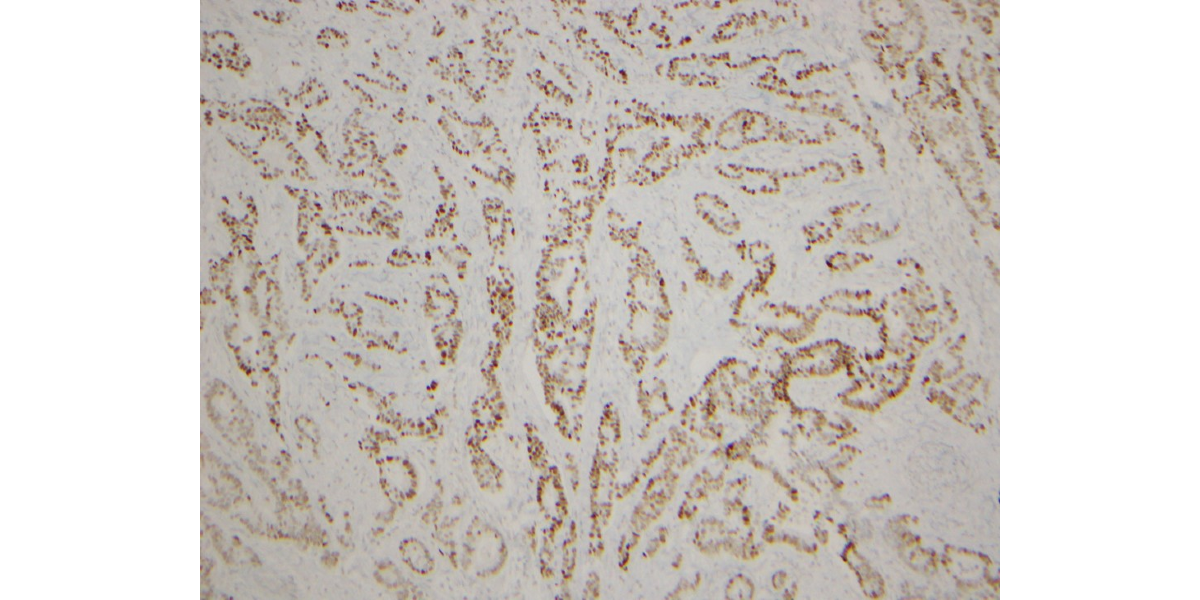
TTF-1 (thyroid transcription factor-1) positive.

Evaluation of the permanent section was consistent with metastatic adenocarcinoma. Immunohistochemical studies were performed and revealed the neoplastic cells to be positive for CK7 (cytokeratin 7) and TTF-1 (thyroid transcription factor-1) consistent with lung origin (Figures 2-5).

The patient was referred to oncology and was found to have stage IV adenocarcinoma of the lung with extensive bony metastasis and involvement of the adrenals, pelvis, and parietal lobe. Excision of the abdominal mass by plastic surgery revealed similar histology.

## Discussion

Cutaneous metastases develop in 0.7% to 9% of patients with cancer. In men, the most frequent sources of metastases are the lung (24%), colon (19%), melanoma (13%), and oral cavity (12%). In women, the most frequent sources of metastases are the breast (69%), colon (9%), melanoma (5%), ovaries (4%), and lung (4%) [1]. Cutaneous metastasis is rather uncommon and has been reported to occur in approximately 5% of all cancer patients [2]. In lung cancer, it is usually a sign of late disease, and concomitant poor prognosis, with a median survival of 3.9 months [3]. In a recent retrospective study of 2130 patients with nonsmall cell carcinoma, only 2.8% had cutaneous metastasis at the time of diagnosis [3]. Our case describes an even rarer occurrence in which cutaneous metastasis manifested as the presenting sign of an underlying malignancy.

Despite the rarity, dermatologists should always consider cutaneous metastasis in their differential for solitary nodules. Our case demonstrates the characteristic lesion of a cutaneous metastasis, which has been described as a flesh-colored or violaceous, nonpainful nodule [2]. The lesion also was located in a high-risk area, the scalp, which makes up 6.9% of cases of cutaneous metastasis [4]. A study of 398 Taiwanese patients with scalp malignancies reported that other than squamous cell carcinoma and basal cell carcinoma, metastatic cutaneous tumors were most common, making up 12.8% of cases. Lung cancer was the leading primary lesion, causing almost a quarter of the cases [5].

This case also demonstrates the critical importance of performing a biopsy and establishing a firm diagnosis prior to initiating MMS. One recent study reported that in 450 patients presenting for MMS with a clinical diagnosis only, 13 of the diagnoses changed following examination of the Mohs debulk specimen. In these patients, a skin biopsy would have changed the management of 9 patients (1%). In 6 of these cases, MMS would not have been performed due to precancerous or benign histology, and in 3 cases, MMS would have been expedited due to the histological presentation of squamous cell carcinoma rather than basal cell carcinoma [6].

In summary, it is rare for an underlying malignancy to present as a cutaneous metastasis. Despite this, it is essential for dermatologists to recognize the classic features and have a high index of suspicion. A timely biopsy can significantly expedite definitive diagnosis, which was delayed in our case by 3 months, potentially impacting the long-term prognosis. Confirmation of a clinical diagnosis with a biopsy prior to MMS can avoid unnecessary procedures and may reveal a more serious pathology. In situations of histologic ambiguity on frozen sections, diagnostic certainty is paramount prior to initiating MMS.

## Abbreviations

CK7: cytokeratin 7
MMS: Mohs micrographic surgery
TIF-1: thyroid transcription factor-1
